# More Than Brothers

**Published:** 1891-08-22

**Authors:** 


### More than Brothers.

We have no need to preach kindness to animals—certain animals, that is—in the monkey-ridden district of Bengal and Western Nepaul. Taught to look upon special species of the brute creation as sacred, the worshippers of Brahm spare neither expense, trouble, nor reverence for what are called the "Zoological demi-gods" of their land.

In Hindostan alone, according to Sir Emerson Tennent, the produce of about ten per cent. of all the stipends of a most charitable population of one hundred and sixty millions, is consecrated to the support of lazy or mischievous brutes. The—what is called—Dheva-Ghee, that is, purveyance system for necessitous animals, comprises some forty or fifty hospitals, and several hundred food dispensaries.

We learn from *Zoological Sketches* (published by Lippincott and Co.), that "in the Bengal Presidency, cows and monkeys enjoy the freedom of several wealthy cities—are permitted to camp in the streets, and help themselves to whatever garbage and surplus fruit the market affords . . . . The *mahakhunds* (literally, 'big yards'), or monkey almhouses, are found near every town and larger village;" these are "special establishments where no low-class monkeys need apply; sick and decrepid honumans and rhesus-baboons are tenderly nursed in several well-appointed hospitals that derive their resources from stipends or pious contributions" from wealthy Buddhists, as well as Brahmins, who thereby secure to themselves a local immortality of glory.

"The baboon-asylum of Bhonaghir, near Hyderabad, feeds about two hundred regulars and fifty or sixty occasional guests, the latter being visitors from the river-bungalows, whose summer vegetation they generally prefer to the somewhat arid neighbourhood of the *mahakhund*." The rule of life observed in these establishments is as follows: "Breakfast is at eight a.m. sharp, but the *dhevadar* beats no gong; his boarders are sure to be on hand. The menu consists of rice, turnips, panicum (a sort of millet), pumpkins, and, now and then, a bushel of figs, served in a pile on the floor, between two troughs of water. As soon as the gate opens, the guests crowd in, the old sachems first, the stout squaws a good second." Anxiously they await the *dhevadar*, and when he appears with a bucket full of good things, "they charge him with a rush, empty his bucket, clamber all over him in search of hidden sweets, and often use him as a jumping-board as they chase each other round the yard. In about four minutes from the first creaking of the gate, the provisions are generally disposed of—provisionally, at least, Providence having provided each baboon with a cheek-pouch of such elastic capacities that a day's rations can be stowed away in one cheek. . . . . A fat old poucher, both cheeks distended with millet, and his four fists full of good extras, will retire into a corner, and growl viciously at the wistful look of a starved youngster. Woe to the low-caste monkey who should attempt to glean the crumbs of their feast. They charge him like bull-dogs, and some one of them at the gate does not fail to take him across the knee, and search his cheek-pouches before dismissing him."

Set a beggar on horseback, &c., says the old adage; the baboons thus well treated speedily show airs—putting themselves in man's place, and man in theirs. As an experiment to try their tempers, the major-domo of a monkey-asylum was induced by much persuasion to postpone the usual hour of the morning meal for an hour and a-half; the consequences fully justified the reluctance of the official to do so as we shall see. "A few minutes after eight, the young baboons became fidgetty, and some of the elders, after strutting up and down in sullen silence, walked to the gate, and began to shake the latch-handle gently at first, but, by and by, with fierce impatience. Then, stepping back, the sachems held a council of war, chattering at each other with protruding lips, and grunting indignantly whenever they looked at the still unopened gate. . . Seeing a man approach from the direction of the village, they gathered round him, evidently in hopes of entering the gate in his wake; but when he pursued his way they vented their displeasure in howls. . . . After another consultation they tried the gate once more, scrutinised the smooth masonry of the wall, and then made for a high tamarind-tree that overlooked the yard of the *mahakhund*. With some difficulty, but with grim resolution," the fat sages of the crowd, not so agile as in their youthful days, clambered to the topmost branches, where they began to "challenge their landlord, with louder and louder claims, rising at last to yells that must have been heard in the distant river-plantations, for, soon after, a deputation of bungalow-baboons came hastening up the rocks, and joined in the chorus before they could possibly have ascertained the cause of the uproar." Lastly, boys appeared upon the scene, and, with their customary obliging readiness to augment anything in the shape of a row, joined manfully in the noisy fray. The "fortissimo yells from the tree-top" being thus aided and abetted by the youthful village, the *dhevadar* hastily capitulated, and opened the door.

In another mahakund, devoted to honumans and other pious paupers of the upper classes, "the guests, under a similar provocation, behaved with more dignity" of demeanour—that is, for, in this case, the elders whiled away the time of waiting—and of their wrath—by taking it out of their youngsters.

". . . towards low-caste animals the honumans show all the intolerance of the bhunder-baboon; sucking-pigs coming within reach of their long arms are often grabbed, and flung through the air with a suddenness that leaves the squealer no time for a protest until he lands sprawling on the other side of the fence. In hospitals for promiscuous animals race-prejudices are, of course, out of place, and, under such circumstances, the honuman communes with his fellow-monkeys on more familiar terms, and often behaves with great kindness—still, however, with a certain condescension, like a Church of England divine in the presence of dissenting ministers."

Like all our over-petted favourites, the inmates of these asylums become frightfully spoiled by indulgence. "In the neighbourhood of populous cities the pupils of a *mahakhund* are exposed to grievous temptations; pious visitors too often surfeit them with sweetmeats," for instance, "the honuman home, hear Allahabad, is burdened with a number of pensioners who are almost too plethoric"—from over-feeding—"to walk, and seem to suffer all the horrors of dyspepsia. Such invalids are the objects of special solicitude, their sufferings being considered as an illustration of the proverbial trials of the just; but in times of scarcity their lot becomes truly pitiful. . . . In the late famine remains were to be seen near Ghuyapor of a baboon institute"—abandoned for lack of necessaries to keep it up—"and its former pampered inhabitants peopled the adjacent woods, reduced to the sad necessity of working for a living, gathering berries and rolling logs in search of coleopterous insects. The youngsters" —with the love of youth for change—"seemed to enjoy their occupation, but the old dyspeptics worked with groans, like the exiled aristocrats, after the French Revolution!" During the well-remembered famine of 1878-79 we are told that "bands of destitute monkeys roamed the country in search of backshish . . . appealing to the charity of strangers . . . even the jugglers had to discharge their dancing macaques . . . and the poor things used to dance on the highway whenever they met a human being," the *Benares Gazette* giving a touching description of a scene at the pier of the boat-bridge, where two of the Terpsichoreans waltzed around a blind beggar, now and then approaching him with beseeching squeaks."

With a final quotation from the interesting account of our more than brothers—as the Brahmins may truthfully style their over-indulged baboons—we must draw to a close our gossip about these gentry.

"An influx of high-caste monkeys has begun to gravitate towards the larger cities . . . " where "while there is flour in the barrel of the true believer there is always bread for the sacred baboon." The marvellous mimics are becoming quite town bred, so to say, and "have begun to take an abstract interest in human affairs. They will gather around a ranting quack, a revivalist, or a broken-down buggy, without any direct view to backshish. If a number of people run toward the scene of an accident, the monkeys race after them like dogs; if the Brahmins get up a pageant, the baboons join in the procession! They take a curious delight in pressing their snub noses against the shop windows of the European merchants, and examine the various novelties with a critical squint. . . . A Delhi bhunder-monkey attracted attention by parading the streets" attired "in two gaudy shawls, evidently not a legal acquisition, as his bad conscience made him take to his heels whenever anybody so much as pointed towards his dry-goods."

On the whole this cursory peep into the life of the pampered baboon gives one an eerie feeling of apprehension as to what, in the end, will be the result if such curiously imitative creatures continue to be so highly trained and foolishly petted.

